# Comparative Analysis Based on Physiological and Transcriptomic Data between Juvenile and Adult Tree Peony (*Paeonia delavayi*)

**DOI:** 10.3390/ijms241310906

**Published:** 2023-06-30

**Authors:** Xiaoli Zhai, Yan Feng, Xiuxin Zhang, Xianfeng Guo

**Affiliations:** 1 College of Forestry, Shandong Agricultural University, Tai’an 271018, China; zhaixil@outlook.com (X.Z.); ifirefly2022@163.com (Y.F.); 2 Key Laboratory of Biology and Genetic Improvement of Horticultural Crops, Ministry Agriculture and Rural Affairs, Institute of Vegetables and Flowers, Chinese Academy of Agricultural Sciences, Beijing 100081, China

**Keywords:** *Paeonia delavayi*, juvenility, physiology, transcriptome analysis, *SPL*

## Abstract

A long juvenile period limits the breeding process of many woody plants including tree peony. To investigate the physiological and transcriptomic differences between juvenile and adult plants of tree peony and to explore the key *SQUAMOSA PROMOTER BINDING PROTEIN-LIKE* (*SPL*) genes, which are vital in age-dependent pathways, 1-year-old and 3-year-old *Paeonia delavayi* plants were used to compare the relevant physiological parameters and transcriptomic profiles of the leaves in two phases of plants. The results of the physiological parameters showed that the starch content in the leaves of adult plants remained unchanged and that the soluble sugar content significantly increased compared with those in the juvenile plants. In terms of plant hormones, the contents of cytokinin-like hormone (N6-isopentenyladenine (iP)) and jasmonic acid (JA) significantly decreased, whereas the contents of auxin (indole-3-acetic acid, IAA), abscisic acid (ABA), cytokinin-like hormone (N6-isopentenyladenenosine (iPR)), and ethylene precursor (1-aminocyclopropane-1-carboxylic acid, ACC) showed no statistic difference. Transcriptome sequencing results showed that there were 194 differentially expressed genes (DEGs) between juvenile and adult plants, including 171 up-regulated DEGs and 23 down-regulated DEGs. Circadian rhythm, plant hormone signal transduction, and sugar metabolism were closely related to the juvenile-to-adult transition in *P. delavayi,* involving a total of 12 DEGs. In addition, a total of 13 *SPL* genes were identified in the transcriptome data, but only *PdSPL10* (c71307.graph_c0) was differentially expressed. It was further validated via qRT-PCR analysis, indicating that *PdSPL10* might be a key gene regulating the process of juvenile-to-adult in *P. delavayi*. Based on the above results, a hypothetical transcriptional network regulating juvenile-to-adult transition and flowering in *P. delavayi* was proposed. These findings provide a reference for understanding the mechanism of juvenile-to-adult transition in tree peony.

## 1. Introduction

Tree peonies usually refer to a group of shrubby plants in genera *Paeonia*. There are nine wild species, specifically indigenous to China [1]. And up to now, thousands of tree peony cultivars have been produced. These plants come in rich colors and multiple flower types, and breeding efforts are still necessary for diversification [2]. However, as deciduous shrubs, they have as long as 4~6 years of a juvenile period before flowering [3]. This relatively long juvenile period seriously hampers the breeding process. Exploring the multifaceted differences between juvenile and adult plants not only helps to understand the inner regulation mechanism responsible for juvenile-to-adult transition but also helps to hunt for the key genes, which will ultimately contribute to accelerating the breeding process.

During the juvenile-to-adult transition in plants, both physiological and molecular changes occur. Physiologically, carbohydrates, endogenous hormones, and other indicators in plants are closely related to this process [4,5,6]. Starch and soluble sugars are the main carbohydrates in plants that act as energy and signal substances that affect plant growth and flowering, and their contents vary with developmental periods [4,7,8]. Endogenous hormones are also important regulatory substances in plants, and their contents in perennial woody plants correspondingly change during the juvenile-to-adult transition. The hormones involved in this process mainly include gibberellic acid (GA), auxin (indole-3-acetic acid, IAA), abscisic acid (ABA), cytokinin (CTK), ethylene, jasmonic acid (JA), and so on [5,9,10]. In some woody horticultural plants such as *Pyrus* and *Malus hupehensis,* the contents of various hormones in juvenile and adult leaves increased or decreased to different degrees [5,11]. To sum up, previous studies have showed that different plants have different change patterns with respect to the contents of carbohydrates and endogenous hormones in the juvenile and adult leaves. So far, how the contents of the above substances differ between juvenile and adult leaves of tree peony has been infrequently reported.

With the rapid development of high-throughput histology technologies in recent years, studies on the molecular aspects about the juvenile-to-adult transition and flowering in plants have been intensified [5,11,12]. RNA-Seq is a deep sequencing technology with high precision and efficiency and is a powerful analysis tool for species lacking reference genomic information [13,14]. In revealing plant flowering mechanisms related to phase transition, significant differences in hormone biosynthesis and signal transduction and photosynthesis have been revealed in juvenile and adult leaves of woody plants such as *Pyrus* and *Rosa chinensis* by comparing RNA-Seq data [5,12]. Moreover, RNA-Seq was used to screen some key factors related to the juvenile-to-adult transition and flowering, such as *SQUAMOSA PROMOTER BINDING PROTEIN-LIKE* (*SPL*); *SUPPRESSOR OF OVEREXPRESSION OF CONSTANS 1* (*SOC1*); floral meristematic tissue characteristic genes; and the transcription factors involved in IAA signal transduction, GA signal transduction, and so on [5,12]. Among these key factors, *SPL* is a noteworthy key factor in the age pathway (miR156/*SPL* module), a major regulatory pathway that regulates the juvenile-to-adult transition and flowering [15,16,17,18]. Significant progress have been made on *SPL* in both model plants and some woody plants. This gene family has different members in different species, with 16 in *Arabidopsis thaliana* [19], 31 in maize [20] and 15 in tomato (*Solanum lycopersicon*) [21]. These *SPL* members play various roles in plant growth and development, mainly involving in plant vegetative phase transition, flower development, leaf development, stress response, and other regulatory processes [16,22,23,24,25,26]. However, the most typical function of *SPL* is promoting the juvenile-to-adult transition and flowering through the miR156/*SPL* module [16,27]. In *Arabidopsis*, the expression of *SPL* increases with age, facilitating the juvenile-to-adult transition and flowering [15,27]. In *Pyrus*, key SPL transcription factors that play a role in the juvenile-to-adult transition and flowering have now been screened [5].

Regarding the juvenile-to-adult transition and flowering process related to tree peony, RNA-Seq analyses have been performed in recent years [28,29,30]. It was found from these reports that plant hormones and circadian rhythm are closely related to this process in tree peony (*P. ostii*, *P. suffruticosa*) [28,29,30]. Candidate genes were revealed to take part in the process [3,4,29,31]. For example, *PsFT* in *P. suffruticosa* was found valuable in shortening the juvenile period of tree peony as overexpression of this gene promoted early flowering in *Arabidopsis* [3]. For another, *PdSPL9* in *P. delavayi* was also found to play a positive regulatory role in this process in a similar ectopic expression experiment, and overexpression of this gene shortened the juvenile period and promoted flowering in transgenic *Arabidopsis* [4]. Moreover, from the RNA-Seq database of *P. suffruticosa*, 16 *PsSPL* genes were identified and 6 of them were proved to be closely related to age via a qRT-PCR analysis [31].

Taken together, some progress has been made on the issue of the juvenile-to-adult transition and the flowering process in tree peony. But, most of these existing studies were mainly about *P. ostii* or *P. suffruticosa*, the congeneric species to *P. delavayi*. Comparative physiological and transcriptomic analyses in *P. delavayi* leaves between juvenile and adult plants have not yet been performed. Therefore, this study was conducted under the aims (1) to reveal the physiological and transcriptomic differences between the leaves of juvenile and adult plants and (2) to explore the key *SPL* gene potentially regulating the juvenile-to-adult transition in *P. delavayi*. The results would provide a clue from a new perspective and would help to reveal the possible regulatory mechanism of the juvenile-to-adult transition.

## 2. Results

### 2.1. Physiological Changes between Juvenile and Adult Plants

**Figure 1:**
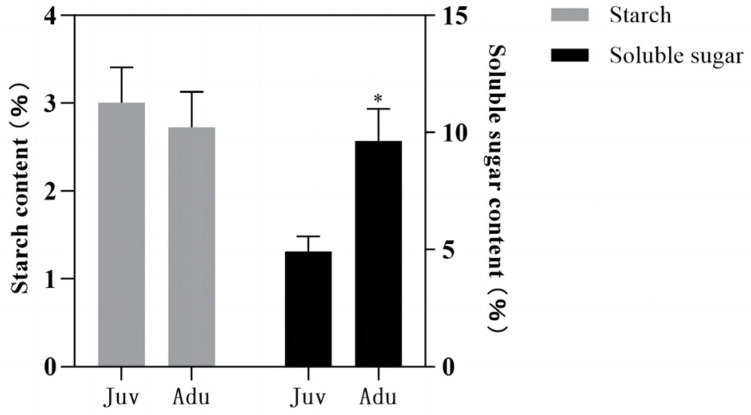
The contents of starch and soluble sugar in juvenile and adult plants of *P. delavayi*. Data were expressed as the mean ± standard error (SE) for three biological replicates. Asterisks (*) indicated Student’s *t*-test significant differences (*p* < 0.05).

Compared with that in juvenile plants, there was no significant change in starch content in adult plants, while the soluble sugar content significantly increased, with only 4.91% in juvenile plants and up to 9.63% in adult plants (Figure 1).

**Figure 2:**
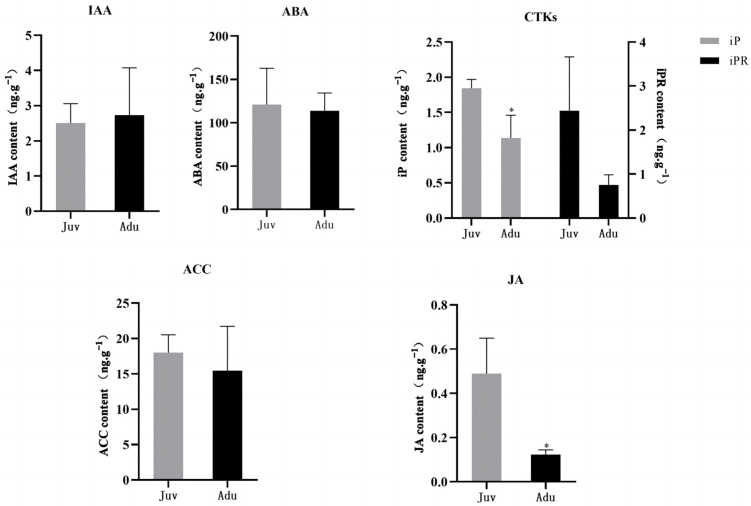
The contents of hormones in juvenile and adult plants of *P. delavayi*. Data were expressed as the mean ± standard error (SE) for three biological replicates. Asterisks (*) indicated Student’s *t*-test significant differences (*p* < 0.05).

The active GAs (GA1, GA3, and GA4), IAA, ABA, CTKs (cZ, tZ, cZR, tZR, iP, and iPR), ACC, and JA were determined in this study (Figure 2). However, GAs as well as cZ, tZ, cZR, and tZR in CTKs were not detectable, probably due to their trace levels or technology limit. Among all the detectable hormones, the contents of CTKs-iP and JA exhibited significant difference (*p* < 0.05). The CTKs-iP content in adult plants was 1.14 ng·g^−1^, significantly lower than the 1.84 ng·g^−1^ in juvenile plants. The content of CTKs-iPR in adult plants was only about 1/3 of that in juvenile plants, although there was no significant difference between the two types of plants. Similarly, the JA content in adult plants was only 0.12 ng·g^−1^, even less than one fourth of that (0.49 ng·g^−1^) in juvenile plants. As for other detectable hormones, their content did not displayed statistic difference.

### 2.2. Differentially Expressed Genes (DEGs) between Juvenile and Adult Plants and Enriched Pathways of DEGs

#### 2.2.1. Transcriptome Sequencing Assembly and Annotation

A total of 87.30 Gb clean data was obtained after sequencing the cDNA libraries of two groups of samples, juvenile (J) and adult (A), and the clean data of each sample reached 12.78 Gb with the percentage of Q30 bases at 92.34% and above, indicating that the data obtained by sequencing were of high quality and could be used for subsequent analysis. A total of 105,994 unigenes were obtained after assembling the obtained high-quality sequencing data, and the N50 of unigenes was 1116, with high assembly integrity.

The annotation statistics of the unigenes in the main nine databases was shown in Table S1 of Supplementary Materials S1. A total of 37,128 unigenes were annotated, while the remaining unigenes were not able to be identified, which implicated that these genes in *P. delavayi* might be novel genes and required further exploration.

#### 2.2.2. Screening of DEGs and Enrichment Analysis of DEGs

A total of 194 DEGs were screened between the two groups of samples, including 171 up-regulated genes and 23 down-regulated genes. Among them, the 28 DEGs with a fold change (FC) above 10 were all up-regulated expression, including 19 annotated genes and 9 unannotated genes (Table S2, in Supplementary Materials S1). The annotated DEGs mainly play roles in transportation and metabolism of both carbohydrates and amino acids, as well as in translocation, defense response, and signal transduction, indicating that the transportation and metabolism of substances become enhanced when tree peony plants enter into adult phase. As for the unannotated genes, their functional enrichment pathways are unknown, implying the possibility that other changes occur during juvenile-to-adult phase.

Furthermore, DEGs related to flowering were searched using the functional annotations and a total of five DEGs were selected: *PdFUL* (c74517.graph_c0), *PdCOL* (c83958.graph_c0), *PdFPF1* (c85008.graph_c0), *PdLHY* (c89148.graph_c0), and *PdSPL10* (c71307.graph_c0) (Table 1). These five genes, positive in flowering process according to documents, unexceptionally showed up-regulated expression pattern, with an FC of 3.25~19.60.

**Figure 3:**
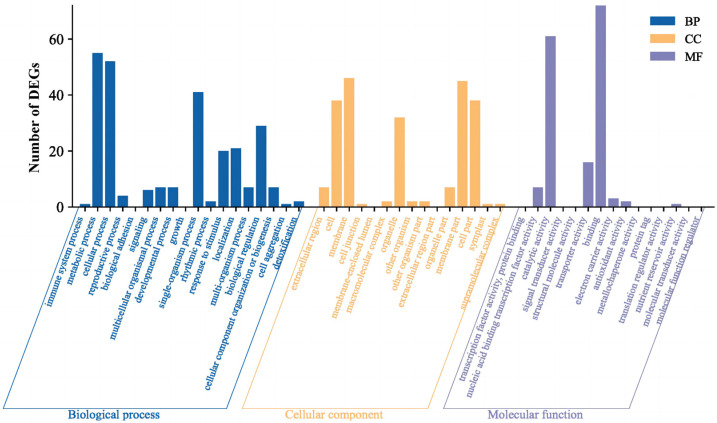
Annotation statistics of GO second-level nodes of the DEGs. The horizontal axis marks the three modules of GO results: biological processes, cellular components, and molecular functions. The vertical axis represents the number of DEGs in each GO term.

The 194 DEGs obtained were subjected to a GO enrichment analysis based on their GO term. As a result, 142 DEGs are annotated in the GO database to three domains: biological processes, cellular components, and molecular functions (Figure 3). At the *p* < 0.005 level, oligopeptide transport, red or far-red light signaling pathway, trehalose biosynthetic process, cellular response to red or far-red light, response to gibberellin, detection of stimulus, integral component of membrane, phosphoric diester hydrolase activity, and so on were significantly enriched.

**Figure 4:**
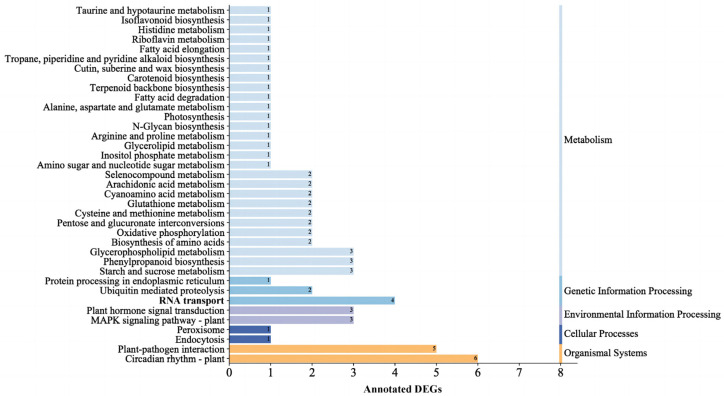
KEGG classification of DEGs. The vertical axis refers to the name of the KEGG metabolic pathway, and the horizontal axis refers to the number of DEGs annotated to the pathway.

The 194 DEGs were further subjected to a KEGG pathway enrichment analysis. As shown in Figure 4, a total of 53 DEGs were mapped to 37 pathways (Supplementary Materials S2). Among these pathways, Circadian rhythm–plant (ko04712) and plant hormone signal transduction (ko04075) were found strongly associated with juvenile-to-adult transition and flowering (*p* < 0.05) (Table S3, in Supplementary Materials S1). In the circadian rhythm–plant pathway, a total of six light signaling-related DEGs were screened (Table 2). These DEGs were enriched at LHY (K12133), CO (K12135), and CDF1 (K16222), all with up-regulated expression (Figure S1, in Supplementary Materials S1). In the plant hormone signal transduction pathway, three DEGs were screened and they were mainly enriched in the pathways of auxin and gibberellin signaling (Figure S2, in Supplementary Materials S1, Supplementary Materials S2). All these DEGs were up-regulated (Table 2).

### 2.3. SPL Genes Expression Analysis

**Figure 5:**
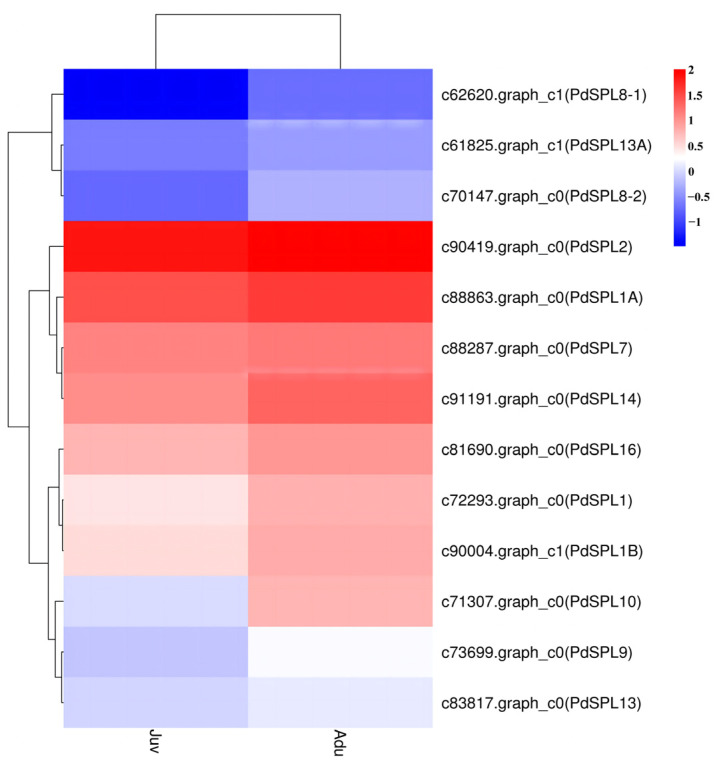
Cluster analysis of *SPL* genes. The color in the heatmap indicates the expression level of the gene in each type of samples and the expression values are Z-score values transformed from log10-based expression values (Table S4, in Supplementary Materials S1).

SPL transcription factors play an important role in the age-dependent flowering pathway [32]. A total of 13 *SPL* genes were found in this study. They are, respectively, *PdSPL1* (c72293.graph_c0), *PdSPL1A* (c88863.graph_c0), *PdSPL1B* (c90004.graph_c1), *PdSPL2* (c90419.graph_c0), *PdSPL7* (c88287.graph)_c0), *PdSPL8*-1 (c62620.graph_c1), *PdSPL8-2* (c70147.graph_c0), *PdSPL9* (c73699.graph_c0), *PdSPL10* (c71307.graph_c0), *PdSPL13* (c83817).graph_c0), *PdSPL13A* (c61825.graph_c1), *PdSPL14* (c91191.graph_c0), and *PdSPL16* (c81690.graph_c0). Their expressions were all up-regulated in adult plants (Figure 5, Table S4, in Supplementary Materials S1), and only the expression of *PdSPL10* produced a significant difference (FDR < 0.05, FC ≥ 2), with its expression in adult plants being 5.64-fold higher than that in juvenile plants.

**Figure 6:**
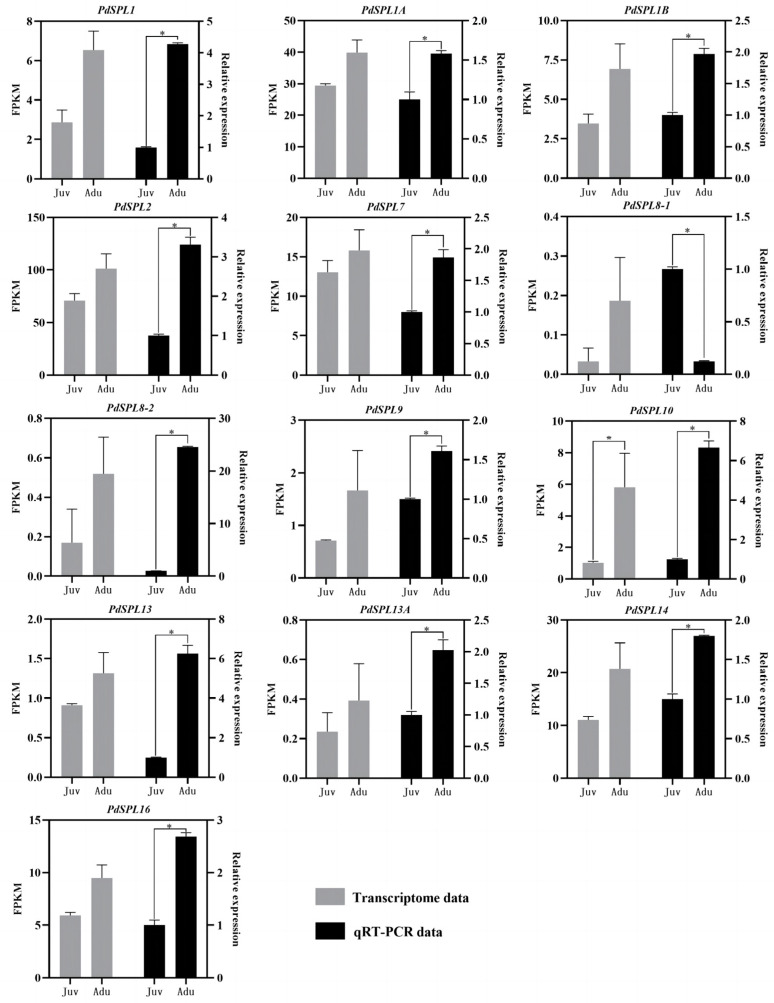
Expression of *SPL* gene in both transcriptome analysis and qRT-PCR analysis. Data were expressed as the mean ± standard error (SE) for three replicates. Transcriptome data were derived from three biological replications; qRT-PCR data were from three technical repetitions. Asterisks (*) indicated false discovery rate (FDR) < 0.05 and fold change (FC) ≥ 2 or Student’s *t*-test significant differences (*p* < 0.05).

qRT-PCR verification results showed that except for *PdSPL8-1*, the expression levels of the other 12 *SPL* genes in adult plants were higher than those in juvenile plants, which was basically consistent with the transcriptome sequencing results (Figure 6). However, the fold difference of each *SPL* gene between juvenile and adult plants was different, although a statistical analysis of the qRT-PCR data showed that the expression of each *SPL* gene differed significantly between the two phases. Among them, the differences of the *PdSPL8-2*, *PdSPL10*, and *PdSPL13* genes were 24.54, 6.66, and 6.24 times, respectively. *PdSPL10* was the only differentially expressed *SPL* gene screened from the transcriptome data.

Combining the transcriptome data and qRT-PCR results, it was inferred that at least *PdSPL10* might play an important role during the juvenile-to-adult transition in *P. delavayi*.

## 3. Discussion

### 3.1. Sugar Signaling (Possibly byT6P Pathway) May Mediate Juvenile-to-Adult Transition in P. delavayi

Carbohydrates are associated with the juvenile-to-adult transition in plants [4,33]. Starch and soluble sugar are two main forms of carbohydrates present in plants. The starch content in adult plants was found to be statistically unchangeable, but the sugar content in adult plants in *P. delavayi* was significantly higher than that in juvenile plants. Regarding the results of starch content, it differs from that in a previous study where the starch content in adult plants of *P*. *delavayi* was significantly higher than that in juvenile plants [4]. This difference may be caused by the fact that starch content varies with growth stages of the plant [34,35]. In a previous study, the leaves were collected in April; whereas in this study, the leaves were collected at the end of May during the flowering period. Plants need a lot of sugar for flowering; thus, starch will be converted into sugar under the action of amylase for direct use by plants [36]. This might partly explain why the starch content was seemingly invariable in the adult phase in the present study. Regarding the results of sugar content, it is consistent with previous results in *P*. *delavayi* and in *R. chinensis* [4,12]. Soluble sugar not only can provide energy for flowering but also can act as an endogenous signal to promote the juvenile-to-adult transition in plants [7,8]. Therefore, we speculate that soluble sugar with high quantity in adult *P. delavayi* plants might serve as an energy source for flowering, and it also might trigger the juvenile-to-adult transition in *P. delavayi*.

The pathway of starch and sucrose metabolism was found among the predominant enriched pathways during the juvenile-to-adult transition in *R. chinensis* and *M. hupehensis* [11,12]. In the present study, the pathway of starch and sucrose metabolism, with a *p* value of 0.29, is not ranked at the top of the list in the KEGG enrichment analysis. However, we noticed that the three DEGs enriched in this pathway were all up-regulated (Figure S3, Table S5, in Supplementary Materials S1), and this trend can partly interpret the increase in sugar content and the juvenile-to-adult transition. Firstly, the up-regulation of the DEG c67297.graph_c0 (glucan endo-1,3-beta-glucosidase) may directly promote the increase in downstream glucose content [37]. In other words, the up-regulated of the above DEG would eventually increase the level of glucose, which is the main soluble sugar [38]. This increase in glucose content is in line with the increase in soluble sugar content. Sugar can down-regulate miR156 expression to mediate the juvenile-to-adult transition [8]. Secondly, the other two DEGs, c82355.graph_c0 (trehalose-phosphate synthase, TPS) and c84316.graph_c3 (trehalose-phosphate phosphatase, TPP), which, respectively, lie upstream and downstream from the trehalose-6-phosphate (T6P) pathway, were significantly up-regulated in adult *P. delavayi* plants. This change may cause a series of reaction—conversion of UDP-glucose (UDPG) and glucose-6-phosphate (G6P) to T6P by TPS, subsequent conversion of T6P into trehalose through TPP, and ultimate hydrolyzation into glucose molecules [39,40]. Moreover, T6P functions as an important signaling molecule in plants signaling molecule in plants, and the T6P pathway can affect juvenile-to-adult transition by repressing the miR156-*SPL* module [15,41]. Therefore, it is collectively deduced that the sugar metabolism pathway (especially the T6P pathway) is related to the juvenile-to-adult transition in *P. delavayi*.

### 3.2. Hormone Signaling (Possibly in Interactive Way) May Mediate Juvenile-to-Adult Transition in P. delavayi

Plant endogenous hormones are trace substances in plants and play an important role in regulating plant growth and development [42]. Previously, IAA, GA, CTK, ABA, JA, and ethylene were reported to be involved in the juvenile-to-adult transition [5,15,43,44,45]. For example, GA promotes flowering by degrading the transcriptional repressor DELLA protein and by releasing SPL [9]. For another, high CTK levels were usually characteristic of juvenile tissues [43,44], and JA declined in advance of the juvenile-to-adult transition in maize [45]. In this study, GAs, whether the commonly detectable GA_3_ or other two less reported GA_1_ and GA_4_, was unexpectedly undetectable. We speculated that this might be caused by technological limitations or too trace levels of GA in *P. delavayi* leaves. Among the detectable hormones, the contents of IAA, ABA, and ACC between two phases of samples differed to some extent, but there was no significant difference. By comparison, the contents of both CTKs-iP and JA between two phases were significantly different.

According to KEGG and GO analysis results of the transcriptome data, DEGs were significantly enriched in the plant hormone signal transduction pathway, but no DEG enrichment specifically occurred in either the CTK or JA pathway. Instead, the auxin pathway and gibberellin pathway were included in the enriched pathways, although GAs were undetectable and the IAA content showed no significant difference. This inconsistency between physiological data and molecular data inferred that the influence of plant hormones upon phase change was more likely through the inter-hormones interaction than purely via their respective absolute values [44,46]. The GA signaling pathway was reported to mediate the juvenile-to-adult transition and flowering through the miR156-*SPL* module. In this pathway, the transcriptional repressor DELLA protein interacts with SPL to inhibit the binding of SPL to downstream flowering-related genes and miR172, leading to the inhibition of flowering, while GA can release SPL through the degradation of DELLA, ultimately promoting flowering [9]. In the present study, in the GA signal transduction pathway, one DEG (c41494.graph_c0, *PdSCL3*) was enriched although GAs were undetectable. SCL3 promotes GA signaling by antagonizing DELLA (major growth repressor) and is a positive regulator of GA signaling [47]. The auxin pathway was also previously reported, being enriched in the juvenile-to-adult transition in plants such as pear [5]. In the present study, two DEGs—the auxin response factor 7 (c86094.graph_c1) and auxin response protein SAUR32-like (c53132.graph_c0) genes—are enriched in this pathway. The expression of both DEGs were up-regulated, which can promote cell growth, and plant growth and development. Collectively, these three DEGs deserved further attention. Since there are cross-talk between different hormone pathways [48,49,50], the exact mechanism needs to be further explored.

### 3.3. Circadian Rhythm Pathway May Mediate Juvenile-to-Adult Process in P. delavayi

The KEGG analysis in this study showed that the DEGs were significantly enriched in the circadian rhythm pathway (the photoperiod pathway). This finding was consistent with previous studies on the flowering of *P. suffruticosa* and *Pleioblastus pygmaeus* [30,51]. Meanwhile, the GO analysis in this study also showed similar results, namely, the DEGs were significantly enriched in the cell response to red or far-red light, and in the signaling pathway of red or far-red light. Previous studies have shown that plant circadian rhythm is an endogenous timing mechanism, which can sense light signals such as red light and far-red light through photosensitive cells of phytochrome and cryptochrome [52,53]. Actually, the two enrichment results in this study consistently indicated that circadian rhythm probably plays an important role in the transition from juvenile to adult and flowering in *P. delavayi*. Among the six enriched DEGs in this pathway, except for the DEG c70837.graph_c0 (annotated as zf-B_box domain-containing protein) that has not yet been precisely annotated, the homologous genes of other five DEGs (*PdCOL2*, *PdCDF3*, *PdLHY*, *PdRVE8*, and *PdRVE1*) have been reported in other plants to be involved in growth and the flowering process [54,55].

### 3.4. Age Pathway May Mediate Juvenile-to-Adult Transition in P. delavayi

Apart from the above-mentioned DEGs in the sugar metabolism pathway, circadian rhythm pathway and plant hormone pathway, *SPL*, acting as a pivotal factor in the miR156-*SPL* module to promote the juvenile-to-adult transition, is a key factor in the age-dependent flowering regulation pathway [56]. In this study, the expression of *PdSPL10* in the leaves of adult plants was significantly higher than that of juvenile plants according to an RNA-Seq analysis, with an FC of 5.64. This was consistent with the result that the expression of some key *SPL* genes significantly increased with plant age in the leaves of some woody plants, such as *SPL3* and *SPL9* in *Eucalyptus globulus* and *Populus* × *canadensis* [57]. Recent studies have found that *SPL10* positively regulates the transition from juvenile to adult and flowering [58,59]. Therefore, it was suggested that *PdSPL10* may be associated with the juvenile-to-adult transition in *P. delavayi*. The possible mechanism is as follows: With juvenile-to-adult transition and flowering, *PdSPL10* repressed by miR156 up-regulates their expression, hence positively regulates downstream flowering-related genes (specifically in this study, *PdFUL*) and miR172, and thus eventually promotes the juvenile-to-adult transition and flowering [32]. In the genus *Paeonia*, a previous study revealed that the expression of *PdSPL9* in the buds of 1-, 2-, and 3-year-old *P*. *delavayi* plants increased with plant age and that *PdSPL9*-overexpressed *Arabidopsis* has a shorter juvenile period and earlier flowering time [4]. The results of this study provide new clues in revealing the molecular mechanism of the juvenile-to-adult transition in tree peony from the perspective of the age pathway. However, the function of *PdSPL10* needs further experimental validation.

### 3.5. A Hypothetical Molecular Regulatory Network during Juvenile-to-Adult Transition in P. delavayi

**Figure 7:**
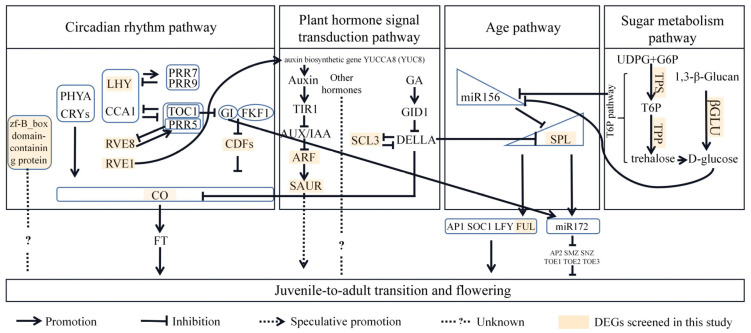
Hypothetical transcriptional regulatory network of juvenile-to-adult transition in *P. delavayi*. In this network, the DEGs are as followed with codes in original transcriptome database in the brackets. *PdCOL2* (c83958.graph_c0, CO), *PdCDF3* (c83340.graph_c1), *PdLHY* (c89148.graph_c0), *PdRVE8* (c84340.graph_c1), *PdRVE1* (c70450.graph_c0), zf-B_box domain-containing protein (c70837.graph_c0); *PdARF19* (c86094.graph_c1), *PdSAUR32* (c53132.graph_c0), *PdSCL3* (c41494.graph_c0); *PdSPL10* (c71307.graph_c0), *PdFUL* (c74517.graph_c0); *PdβGLU* (c67297.graph_c0), *PdTPS* (c82355.graph_c0), *PdTPP* (c84316.graph_c3). The following is a list of abbreviations in this network. PHYA: PHYTOCHROMEA, CRYs: CRYPTOCHROMES, LHY: LATE ELONGATED HYPOCOTYL, CCA1: CIRCADIAN CLOCK ASSOCIATED1, GI: GIGANTEA, FKF1: FLAVIN BINDING, KELCH REPEAT, F-BOX1, CDF: CYCLING DOF FACTOR, CO: CONSTANS, FT: FLOWERING LOCUS T, GA: gibberellin, GID1: GIBBERELLIN INSENSITIVE DWARF1, SCL3: SCARECROW-LIKE3, miR156: microRNA156, SPL: SQUAMOSA PROMOTER BINDING PROTEIN-LIKE, AP1: APETALA1, SOC1: SUPRESSOR OF OVEREXPRESSION OF CONSTANS1, LFY: LEAFY, miR172: microRNA172, AP2: APETALA2, SMZ: SCHLAFMUTZE, SNZ: SCHNARCHZAPFEN, TOE1-3: TARGET OF EAT1-3, UDPG: UDP-glucose, G6P: glucose-6-P, TPS: TREHALOSE-6-PHOSPHATE SYNTHASE, TPP: TREHALOSE PHOSPHATE PHOSPHATASES, T6P: trehalose-6-phosphate. References: [8,9,33,39,41,47,54,55].

Based on the above analysis mainly derived from the transcriptome data and a previous study, we present a hypothetical transcriptional regulatory network of the juvenile-to-adult transition in *P. delavayi* (Figure 7). In this network, we proposed that a total of 14 DEGs associated with different pathways—6 in circadian rhythm pathway (*PdCOL2*, belonging to CO, *PdCDF3*, *PdLHY*, *PdRVE8*, *PdRVE1*, and zf-B_box domain-containing protein), 3 in the plant hormone signal transduction pathway (*PdARF19*, *PdSAUR32*, and *PdSCL3*), 1 in the age pathway (*PdSPL10*); 1 in the downstream of the age pathway (*PdFUL*), and 3 in sugar metabolism pathway (*PdβGLU*, *PdTPS*, and *PdTPP*).

These DEGs are potentially valuable for they enlarged the gene resources in regulating the juvenile-to-adult transition in *P. delavayi*. The function and functional mechanism of these genes need further validation. On this basis, it will eventually facilitate the breeding work of tree peony. Specifically, with these explored gene resources, along with the increasingly mature genetic transformation system of tree peony [60,61], breeding peony varieties with short juvenile period in the future will become possible, just like the early flowering strains of apple (*Malus* × *domestica*) and *Eucalyptus* obtained through genetic engineering [62,63].

## 4. Materials and Methods

### 4.1. Plant Materials

In this study, 30 juvenile plants (1-year-old) and 30 adult plants (3-year-old) of *P. delavayi* were used as experimental materials. These plants were grown at the tree peony planting base of the Institute of Vegetable and Flower, Chinese Academy of Agricultural Sciences (Yanqing Farm, Beijing). Fresh leaves (the third basal compound leaf) were sampled from the juvenile and adult plants during the flowering period in May 2021. The mixture of every 10 leaf samples was regarded as one biological replicate. Thus, each phase of the samples contained three biological replicates, and a total of six samples were acquired. After quick freezing in liquid nitrogen, these samples were then stored in a refrigerator at −80 °C for the determination of physiological parameters, transcriptome sequencing, and gene expression analysis.

### 4.2. Methods

#### 4.2.1. Quantitation of Carbohydrates and Plant Hormones

The contents of starch and soluble sugar were determined using the anthrone colorimetric method [64,65]. Using a SCIEX ExionLC AD system equipped with an AB SCIEX QTRAP 5500 mass spectrometer (AB SCIEX, Framingham, MA, USA), the contents of endogenous hormones were determined via liquid chromatography-tandem mass spectrometry (LC-MS/MS) [66]. The determined hormones mainly included active GAs (GA1, GA3, GA4), IAA, ABA, CTKs (cis-zeatin (cZ), trans-zeatin (tZ), cis-Zeatin riboside (cZR), trans-Zeatin riboside (tZR), N6-isopentenyladenine (iP), N6-isopentenyladenenosine (iPR)), ethylene precursor (1-aminocyclopropane-1-carboxylic acid, ACC), and JA.

All the determinations were performed in triplicates. All data were expressed as mean ± standard error (SE), and the Student’s *t*-test was used for statistical analysis. GraphPad Prism 7.0 (GraphPad Software, San Diego, CA, USA) was used for analysis and image plotting.

#### 4.2.2. Discovery of DEGs and Pathways

The total RNA was extracted from each sample using RNA extraction kits (Aidlab Biotech, Beijing, China) according to the manufacturer’s instructions. After a quality test of the extracted RNA, library construction and transcriptome sequencing were carried out by BioMarker Biotechnology Co. (Beijing, China) using the Illumina Hiseq platform. The sequencing joints and primers in reads were removed from the raw data, and the low-quality data were filtered. Then, the high-quality clean data were used for sequence assembly using software Trinity (https://www.trinityconsultants.com/software), and thereby, unigene sequences were obtained. Then, the unigenes were identified in the databases including NR, Swiss-Prot, COG, KOG, eggNOG4.5, Pfam, TrEMBL, GO, and KEGG.

The unigene expression levels were expressed as normalized fragments per kilobase of transcript per million mapped reads (FPKM). To obtain DEGs between the two groups of samples, DESeq2 was performed. A false discovery rate (FDR, the corrected *p*-value obtained by Wald test) < 0.05 and fold change (FC) ≥ 2 were used as the difference screening criteria. The flowering-related DEGs were further identified according to the functional annotation results.

An enrichment analysis was performed using clusterProfiler (Guangzhou, China) after completing GO and KEGG annotations based on InterProScan (EMBL-EBI, Hinxton, Cambridgeshire, UK) [67] and KOBAS (Peking University, Beijing, China) [68], and enrichment significance was calculated using a hypergeometric test.

#### 4.2.3. Expression Analysis of SPL Genes

*SPL* genes were identified according to the functional annotation results. The expression of the *SPL* gene in the transcriptome was analyzed based on the FPKM values.

To confirm the expression of these *SPL* genes, a real-time quantitative PCR (qRT-PCR) was performed using gene-specific primers (Table S6, in Supplementary Materials S1). *Actin* was selected as the reference gene. For either the juvenile or adult leaf samples, three biological replicates were mixed to extract the RNA. The extraction method was the same as that reported in Section 2.2.2. Then, cDNA was obtained according to the instructions of the Vazyme Reverse Transcription Kit (Vazyme, Nanjing, China). The reaction was performed with a Bio-Rad CFX96™ real-time PCR instrument (Bio-Rad, Hercules, CA, USA) using the following procedure: pre-denaturation at 95 °C for 30 s; 40 cycles of 95 °C for 5 s and 60 °C for 30 s; 95 °C for 10 s, 65 °C for 5 s, and 95 °C for 5 s. All qRT-PCR experiments were performed with three technical repetitions. The relative expression levels of the *SPL* genes were calculated using the 2^−ΔΔCT^ method [69]. All data were expressed as mean ± standard error (SE), and the Student’s *t*-test was used for statistical analysis.

## 5. Conclusions

The soluble sugar content in the leaves of adult plants was significantly increased compared with that of juvenile plants, whereas the starch content remained unchanged. The contents of CTKs-iP and JA were significantly decreased, whereas the contents of IAA, ABA, CTKs-iPR, and ACC (ethylene precursor) remained unchanged. Circadian rhythm, plant hormone signal transduction, and sugar metabolism were closely related to the juvenile-to-adult transition in *P. delavayi*. The expression of the *PdSPL* genes were up-regulated in adult plants compared with that in juvenile plants, among which, *PdSPL10* was differentially expressed and may play a positive regulatory role in the juvenile-to-adult transition in *P. delavayi*. Further, a hypothetical transcriptional regulatory network including 14 DEGs for the juvenile-to-adult transition in *P. delavayi* was constructed.

## Figures and Tables

**Table 1:** Flowering-related differentially expressed genes (DEGs). Both J-FPKM and A-FPKM are the average value of three biological replicates.

ID	Gene Annotation	Gene Name	J-FPKM	A-FPKM	Fold Change
c74517.graph_c0	AP1/FUL-like protein	*PdFUL*	5.55	32.18	5.80
c83958.graph_c0	CONSTANS-LIKE 2	*PdCOL* *2*	16.15	52.53	3.25
c85008.graph_c0	FLOWERING-promoting factor 1	*PdFPF1*	2.73	44.99	16.48
c89148.graph_c0	protein LHY	*PdLHY*	1.97	38.62	19.60
c71307.graph_c0	promoter-binding protein SPL10	*PdSPL10*	1.03	5.81	5.64

**Table 2:** DEGs in circadian rhythm–plant pathway and plant hormone signal transduction pathway.

KEGG Pathway	ID	Gene Annotation	Up/Down-Regulated	Fold Change
Circadian rhythm-plant	c70450.graph_c0	REVEILLE 1	up	4.12
	c70837.graph_c0	B-box zinc finger protein 19	up	4.49
	c83340.graph_c1	Cyclic dof factor 3	up	5.07
	c83958.graph_c0	CONSTANS-LIKE 2	up	3.25
	c84340.graph_c1	REVEILLE 8	up	3.41
	c89148.graph_c0	LHY	up	19.57
Plant hormone signal transduction	c41494.graph_c0	Scarecrow-like protein 3	up	3.08
	c53132.graph_c0	Auxin-responsive protein SAUR32-like	up	2.79
	c86094.graph_c1	Auxin response factor 7	up	2.66

## Data Availability

The sequence data generated for this study can be found in the NCBI and can be found using accession number PRJNA909852 (http://www.ncbi.nlm.nih.gov/bioproject/909852 (accessed on 7 December 2022)).

## Supplementary Materials

The following supporting information can be downloaded at https://www.mdpi.com/article/10.3390/ijms241310906/s1.
