# Supplementary material for: Comparative Analysis Based on Physiological and Transcriptomic Data between Juvenile and Adult Tree Peony (Paeonia delavayi)
Source: Int J Mol Sci. 2023 Jun 30;24(13):10906. doi: 10.3390/ijms241310906 (PMC10341753; doi:10.3390/ijms241310906)
Supplement: Supplementary file 1 [file ijms-24-10906-s001.zip › Supplementary_Material_S1.pdf]

International Journal of
Molecular Sciences

MDPI

Article

Comparative Analysis Based on Physiological and Transcriptomic Data between Juvenile and Adult Tree Peony (*Paeonia delavayi*)

Xiaoli Zhai [1], Yan Feng [1], Xiuxin Zhang [2,*] and Xianfeng Guo [1,*]

1 College of Forestry, Shandong Agricultural University, Tai'an 271018, China; zhaixil@outlook.com (X.Z.); ifirefly2022@163.com (Y.F.)
2 Key Laboratoryof Biology and Genetic Improvement of Horticultural Crops, Ministry Agriculture and Rural Affairs, Institute of Vegetables andFlowers, Chinese Academy of Agricultural Sciences, Beijing 100081, China
* Correspondence: zhangxiuxin@caas.cn (X.Z.); guoxf@sdau.edu.cn (X.G.)

Citation: Zhai, X.; Feng, Y.; Zhang, X.; Guo, X. Comparative Analysis Based on Physiological and Transcriptomic Data between Juvenile and Adult Tree Peony (*Paeonia delavayi*). *Int. J. Mol. Sci.* **2023**, *24*, 10906. https://doi.org/10.3390/ijms241310906

Academic Editor: Zsófia Bánfalvi

Received: 8 June 2023
Revised: 25 June 2023
Accepted: 27 June 2023
Published: 30 June 2023

Copyright: © 2023 by the authors. Submitted for possible open access publication under the terms and conditions of the Creative Commons Attribution (CC BY) license (https://creativecommons.org/licenses/by/4.0/).

Supplementary Figures and Tables

Supplementary Figures

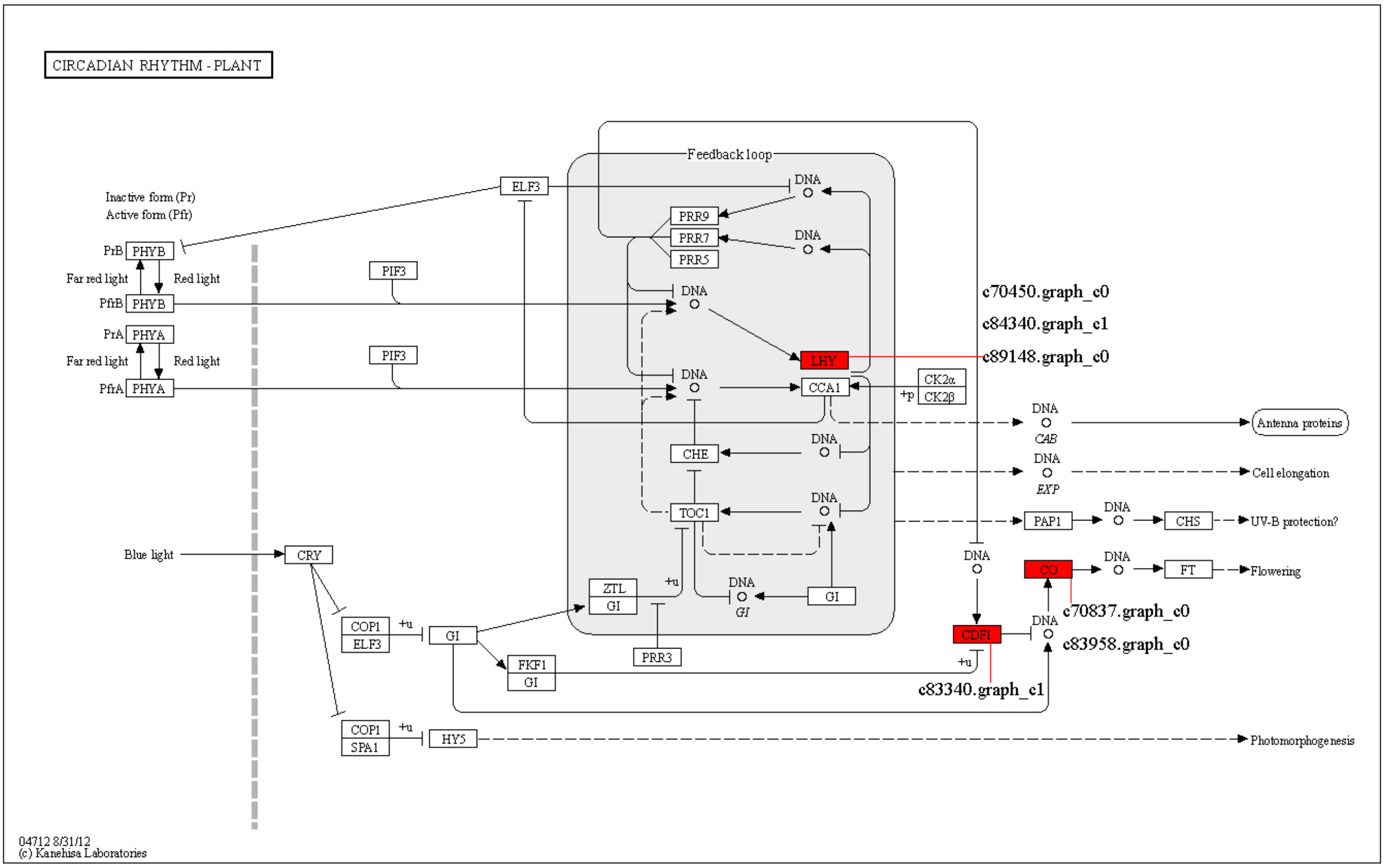

Figure S1. Circadian rhythm-plant pathway.

A total of six DEGs were enriched in this pathway. Red squares represent sites where DEGs are enriched and up-regulated. The red line connects the IDs of the genes enriched at the site.

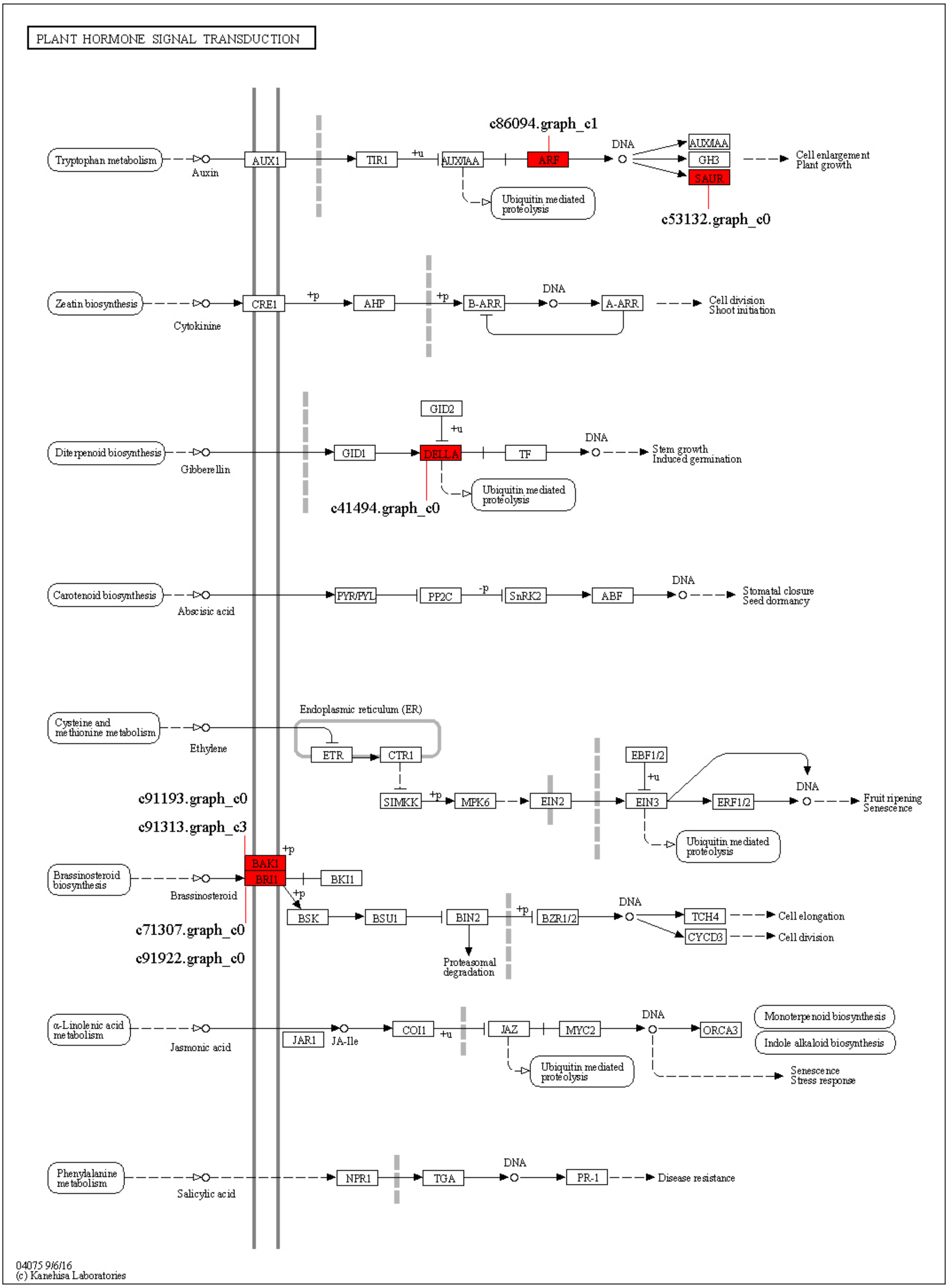

Figure S2. Plant hormone signal transduction pathway.

A total of seven DEGs were enriched in this pathway. Red squares represent sites where DEGs are enriched and up-regulated. The red line connects the IDs of the genes enriched at the site. After combining the annotation results of these seven DEGs in various databases and comparing the results in NCBI (Supplementary 2), we found that the KEGG annotation results of four genes enriched in the BR pathway were incorrect, so these four genes were removed from the phytohormone signaling pathway and these four genes were also removed from the total DEGs enriched by KEGG.

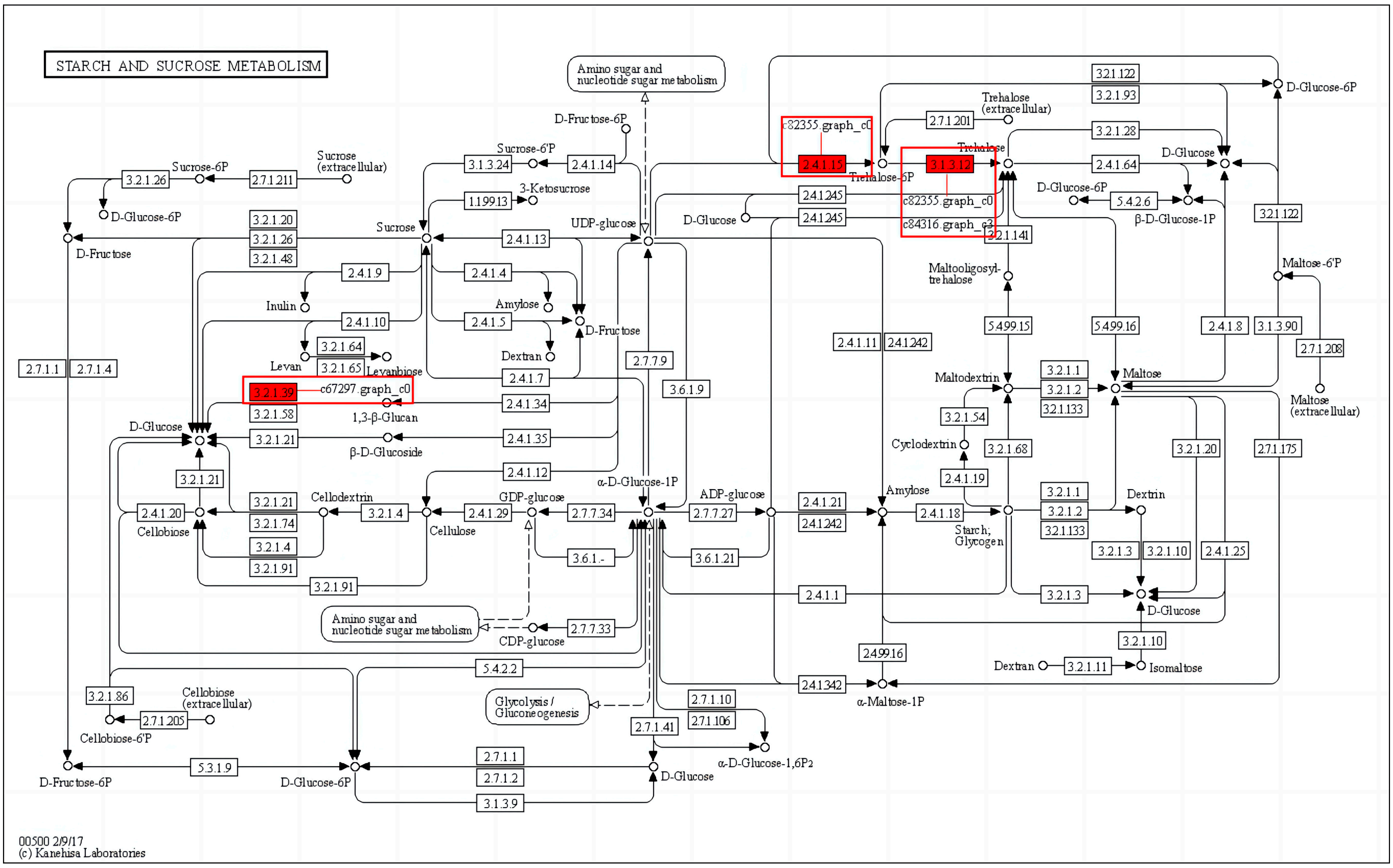

Figure S3. Starch and sucrose metabolism pathway.

A total of three DEGs were enriched in this pathway. Among them, c82355.graph_c0 appears twice. Red squares represent sites where DEGs are enriched and up-regulated. The red line connects the IDs of the genes enriched at the site.

Supplementary Tables

Table S1. Unigene annotated statistics table.

Annotated databases: indicates each functional database; Annotated_Number: indicates the number of Unigene annotated to this database; 300 ≤ length < 1000: indicates the number of Unigene with length greater than or equal to 300 and less than 1000 bases annotated to this database; length ≥ 1000: indicates the number of Unigene with length greater than 1000 bases annotated to this database.

Annotation _Database	Annotated_Number	300 ≤ length < 1000	Length ≥ 1000
COG	7961	2141	4947
GO	27558	9572	12783
KEGG	21350	6919	10867
KOG	21275	7176	9106
Pfam	22891	7384	12292
Swissprot	19554	6069	10859
TrEMBLn	32421	11492	14931
eggNOG	26957	9238	13198
nr	32773	11641	14926
All	37128	13424	15177

Table S2. Information on DEGs with fold change above 10.

"-" is expressed without comment

ID	Gene annotation	J-FPKM	A-FPKM	Fold change
c87423.graph_c0	-	0.01	21.24	2124
c71829.graph_c0	-	0.51	436.58	856.04
c80869.graph_c0	Polygalacturonase-like	0.12	5.61	46.75
c86681.graph_c1	-	0.86	38.78	45.09
c83760.graph_c1	-	0.22	7.82	35.55
c67674.graph_c0	Protein NRT1/ PTR FAMILY 2.11-like	0.45	14.69	32.64
c72370.graph_c0	-	0.61	18.17	29.79
c83409.graph_c1	-	0.42	12.15	28.93
c89148.graph_c0	Protein LHY isoform X3	1.97	38.62	19.60
c87984.graph_c1	B-box zinc finger protein 32	0.35	6.82	19.49
c67674.graph_c1	Protein NRT1/ PTR family 2.11	0.62	10.89	17.56
c69881.graph_c0	Cinnamoyl-CoA reductase 2	0.57	9.73	17.07
c66542.graph_c0	Asparagine synthetase [glutamine-hydrolyzing] 1	1.26	21.23	16.85
c85008.graph_c0	Flowering-promoting factor 1	2.73	44.99	16.48
c82304.graph_c0	F-box protein AFR	1.12	17.98	16.05
c67665.graph_c0	-	34.15	538.78	15.78
c87504.graph_c1	Ribonuclease H	1.68	25.50	15.18
c69385.graph_c0	PREDICTED: umecyanin	5.79	82.81	14.30
c88553.graph_c0	Hypothetical protein F0562_005770	0.52	7.29	14.02
c72622.graph_c0	Uncharacterized protein LOC109022302 isoform X3	13.47	176.78	13.12
c91313.graph_c3	G-type lectin S-receptor-like serine/threonine-protein kinase LECRK1	0.40	5.20	13.00
c84902.graph_c2	Uncharacterized protein LOC107430685 isoform X2	3.54	46.56	12.87
c82465.graph_c1	Ethylene-responsive transcription factor ERF003-like	1.80	22.47	12.48
c77698.graph_c0	Cationic amino acid transporter 5-like	0.64	7.56	11.81
c81753.graph_c0	-	0.22	2.41	10.95
c81112.graph_c0	TIP41-like protein	0.25	2.70	10.80
c90857.graph_c0	Retrovirus-related Pol polyprotein from transposon RE1 OS	0.72	7.47	10.38
c91193.graph_c0	Rust resistance kinase Lr10	6.61	67.05	10.14

Table S3. KEGG-enriched metabolic pathway of DEGs.

The table shows information about the top 20 enrichment pathways, the top two of which are associated with the juvenile-to-adult transition and flowering.

ID	Description	Gene number	DEG number	p-value
ko04712	Circadian rhythm-plant	87	6	5.1441×10^{-6}
ko04075	Plant hormone signal transduction	459	3	0.009
ko00450	Selenocompound metabolism	39	2	0.017
ko00590	Arachidonic acid metabolism	45	2	0.022
ko00430	Taurine and hypotaurine metabolism	9	1	0.045
ko00564	Glycerophospholipid metabolism	157	3	0.061
ko00943	Isoflavonoid biosynthesis	17	1	0.084
ko00460	Cyanoamino acid metabolism	129	2	0.142
ko00940	Phenylpropanoid biosynthesis	260	3	0.149
ko00340	Histidine metabolism	36	1	0.169
ko00480	Glutathione metabolism	153	2	0.185
ko00740	Riboflavin metabolism	40	1	0.186
ko03013	RNA transport	477	4	0.229
ko00062	Fatty acid elongation	53	1	0.239
ko00960	Tropane, piperidine and pyridine alkaloid biosynthesis	53	1	0.239
ko00500	Starch and sucrose metabolism	367	3	0.292
ko00073	Cutin, suberine and wax biosynthesis	68	1	0.296
ko00906	Carotenoid biosynthesis	71	1	0.307
ko00270	Cysteine and methionine metabolism	220	2	0.313
ko04626	Plant-pathogen interaction	735	5	0.326

Table S4. The FPKM, FDR, FC and lg (FPKM) values of *PdSPL* genes in transcriptome data.
Some *SPL* genes with very low expression were not included in the differentially expressed gene screen and no FDR values existed.

ID	Gene Name	J-FPKM	lg (J-FPKM)	A-FPKM	lg (A-FPKM)	FDR	FC
c72293.graph_c0	*PdSPL1*	2.87	0.46	6.57	0.82	0.24	2.28
c88863.graph_c0	*PdSPL1A*	29.46	1.47	39.84	1.60	0.67	1.35
c90004.graph_c1	*PdSPL1B*	3.48	0.54	6.93	0.84	0.25	1.99
c90419.graph_c0	*PdSPL2*	70.99	1.85	101.19	2.01	0.51	1.43
c88287.graph_c0	*PdSPL7*	13.03	1.11	15.79	1.20	1.00	1.21
c62620.graph_c1	*PdSPL8-1*	0.03	-1.48	0.19	-0.73	--	6.33
c70147.graph_c0	*PdSPL8-2*	0.17	-0.77	0.52	-0.28	--	3.06
c73699.graph_c0	*PdSPL9*	0.71	-0.15	1.66	0.22	--	2.33
c71307.graph_c0	*PdSPL10*	1.03	0.01	5.81	0.76	0.03	5.64
c83817.graph_c0	*PdSPL13*	0.91	-0.04	1.31	0.12	0.59	1.44
c61825.graph_c1	*PdSPL13A*	0.24	-0.63	0.39	-0.41	--	1.63
c91191.graph_c0	*PdSPL14*	11.07	1.04	20.75	1.32	0.36	1.87
c81690.graph_c0	*PdSPL16*	5.93	0.77	9.48	0.98	0.38	1.60

Table S5. The FPKM, FDR and FC values of DEGs in the starch and sucrose metabolic pathways.

ID	Gene annotation	Gene Name	J-FPKM	A-FPKM	FDR	FC
c67297.graph_c0	glucan endo-1,3-beta-glucosidase, acidic-like	*PdβGLU*	179.90	1016.73	0.049	5.65
c82355.graph_c0	alpha-trehalose-phosphate synthase	*PdTPS*	0.62	4.10	0.040	6.61
c84316.graph_c3	trehalose-phosphate phosphatase	*PdTPP*	11.41	37.61	0.049	3.30

Table S6. The primers for qRT-PCR.

Genes	Primer sequence (F)	Primer sequence (R)
PdActin	GTCAGCCATTCCATCACCTT	CTTGAGACGACCATCCTTGTT
PdSPL1	TTCCTTCGGCGTTCATTATG	GTCAGCAATGTAGCAGGTTC
PdSPL1A	CTTCTTCACAGGACTCAACTAA	GACTACAGCACACCAATCAT
PdSPL1B	TGTTGTCAGGTTGATGATTGT	TCCTACCAGTGCTCTACAG
PdSPL2	GTTCCAGACCACCTACGATGAC	CACACGCTGCCAAGTTGAAG
PdSPL7	CGCAACGCCTCCTCTAACAA	CGGTGAGAGTTGAGATGGTGATAG
PdSPL8-1	GAGAGGAGGTTCTGGTGAT	GAATAGAGACAATGCTGGAGTA
PdSPL8-2	CGGAGTTCATTCATCTTCATCATT	GTCGTGATATGGTAAGGTCTCT
PdSPL9	GCAATTCTGGACATCCTTCATCA	CACAACTGGAATCGGACACTC
PdSPL10	AGGTGACTCTGTAGCATCTG	GTGAACTCTGTGTACTGTGATAAG
PdSPL13	TACACCCATCAACCAAACAAGACA	AAGGACCCAAAGGCATCAACA
PdSPL13A	GTGACTGGAACCTGCTACA	GCCTTGTTGATGAGTGTGAT
PdSPL14	GTCTCCTTCATCTTCTCCAGTT	AGTCCTGCTTCCTTCAACAT
PdSPL16	TCCATTCTGATCGTGCTCTC	AACCTCCATTCCTAAGCCATT
